# Wanted: Dead or Alive Cells with Propidium Iodide Staining in Liver Tissue

**DOI:** 10.3390/ijms252413521

**Published:** 2024-12-17

**Authors:** Tim Christopher Krapoth, Gina Sophie Henle, Mihrije Avdyli, Berina Bektić, Katharina Maria Schwarzkopf, Larisa Bešić, Stefan Zeuzem, Christoph Welsch, Nico Kraus, Cristina Ortiz

**Affiliations:** 1 Goethe University, Frankfurt University Hospital, Medical Clinic 1, 60596 Frankfurt, Germany; krapoth@em.uni-frankfurt.de (T.C.K.); ginasophiehenle@gmail.com (G.S.H.); mihrije.av@icloud.com (M.A.); bektic@med.uni-frankfurt.de (B.B.); schwarzkopf@med.uni-frankfurt.de (K.M.S.); zeuzem@em.uni-frankfurt.de (S.Z.); welsch@med.uni-frankfurt.de (C.W.); n.kraus@med.uni-frankfurt.de (N.K.); 2 Department of Genetics and Bioengineering, International Burch University, Francuske Revolucije bb, 71210 Sarajevo, Bosnia and Herzegovina; larisa.besic@ibu.edu.ba

**Keywords:** propidium iodide (PI), 4′,6-diamidino-2-phenylindole (DAPI), TUNEL assay, liver pathology, cryosections, dead cell detection, immunofluorescence

## Abstract

This study demonstrates the effectiveness of propidium iodide as a reliable marker for detecting dead or dying cells in frozen liver tissue sections. By comparing propidium iodide staining with the widely used Terminal deoxynucleotidyl transferase dUTP nick end labeling (TUNEL) assay, both methods showed consistent results in disease models such as alcohol-induced fibrosis and Western diet-induced fatty liver. Additionally, propidium iodide was successfully co-stained with other fluorescent markers, like phalloidin (for actin filaments) and antibodies targeting collagen, enabling detailed spatial analysis of dying cells within tissue. This multiplex approach allows for a deeper understanding of tissue organization and cell death localization, particularly in complex conditions like liver fibrosis. Moreover, our results suggest that propidium iodide staining can be applied beyond current models, offering a more accessible and cost-effective alternative to traditional methods, like TUNEL. Furthermore, its integration with other markers enables simultaneous analysis of immune responses and tissue damage, making it a powerful tool for future studies on liver disease and other inflammatory conditions. This technique has the potential to advance research into disease mechanisms and improve the evaluation of novel therapeutic strategies targeting tissue regeneration and inflammation control.

## 1. Introduction

Cell death, such as apoptosis, plays a crucial role in maintaining homeostasis and regulating various biological processes, including immune responses, tissue development and disease pathogenesis [1]. Accurate identification of dying cells is critical to understanding these processes at the cellular and molecular level [2]. One of the most widely used methods for detecting cell death is the use of specific staining techniques that allow researchers to distinguish between living, dying, and dead cells based on specific biochemical and morphological features [3]. However, despite advances in cell death staining protocols, clearly distinguishing between cell death-positive and cell death-negative cells remains a major challenge, especially when analyzing complex tissue environments or immune cell populations [3].

Several established methods are currently used to detect cell death, each with its specific strengths and limitations. One widely used technique is Terminal deoxynucleotidyl transferase dUTP nick end labeling (TUNEL) staining, which identifies DNA fragmentation—a key feature of apoptosis [4]. During cell death, DNA is cleaved, leaving numerous exposed 3′-OH ends [5]. TUNEL staining specifically labels these DNA breaks by incorporating modified nucleotides at the exposed ends, allowing for sensitive detection of apoptotic cells [4]. This method is highly versatile, as it can be applied to both in vitro cell cultures and ex vivo tissue samples, making it suitable for a wide range of experimental models, including complex tissues. Moreover, TUNEL staining can distinguish apoptotic cells from necrotic and viable ones, making it particularly useful for studies aiming to assess apoptosis in tissue sections or entire organs [6]. Despite its broad applicability, TUNEL is often used as a standalone technique and may lack integration with other markers, which can limit its use for in-depth analysis of cellular phenotypes.

Another commonly used method is propidium iodide (PI) staining, a membrane-impermeable dye that selectively stains dead cells or late-stage apoptotic cells [7]. PI binds to DNA by intercalating between the bases and fluoresces upon binding, effectively distinguishing necrotic or late-apoptotic cells from viable ones, as it cannot penetrate intact cell membranes [8]. However, the application of PI is mostly restricted to in vitro studies using cell cultures [9]. Experiments to confirm cell death in tissues using PI are widespread. For example, PI is often used in brain tissue sections in organotypic hippocampal slice cultures to detect cell death [10,11]. However, this method has not yet been described in the liver, which is why we have developed this protocol to quantify cell death in liver cryosections. Dead or dying cells in animal tissues are often analyzed using fluorescence-activated cell sorting [12]. This technique allows dead cells to be labelled with PI, enabling their distinction from healthy, intact cells. Fluorescence-activated cell sorting is a rapid and straightforward method for quantifying cell death. However, it does not provide spatial information, such as the specific tissue zones where cell death occurs, which can be critical for understanding localized patterns of cell damage. In tissue samples, especially in complex and highly vascularized organs like the liver, PI penetration is hindered by the dense tissue architecture and cellular barriers, leading to inconsistent staining results. As a result, PI staining is less effective for ex vivo and in vivo studies, where accurate detection of cell death in tissue is critical for understanding disease processes and tissue responses [13].

The need for unambiguous identification of cell death-positive cells is particularly pronounced in studies with immune cells, cancer, or degenerative diseases [14,15], where, in addition to cell phenotyping, tissue structure, zonation of cells in the overarching matrix and metabolic performance are also important. In such studies, a precise distinction between healthy and dying cells influences both the accuracy of the experiments and the interpretation of the effects of cell death on the surrounding tissue environment. Furthermore, current cell death assays often fail to integrate with other biological markers, limiting their use in comprehensive immunophenotyping and multiplex studies [14].

To overcome the limitations of currently available methods for detecting cell death, there is an increasing need for new methods that combine cell death markers with other molecular signatures using advanced multiplex immunofluorescence technology [15].

Multiplex immunofluorescence techniques allow for the simultaneous detection of cell death alongside other key markers, such as immune activation, fibrosis, or metabolic stress, enabling researchers to explore the intricate pathways involved in liver diseases, e.g., chronic liver disease and liver cirrhosis, hepatocellular carcinoma, or autoimmune hepatitis [3,15,16]. In liver cancer, for example, it is essential not only to detect apoptotic tumor cells, but also to assess the response of surrounding immune cells and stromal elements, which can either support or hinder tumor growth [17]. Similarly, in chronic fibrotic liver diseases, multiplexing techniques can help map the interactions between dying hepatocytes and activated hepatic stellate cells, providing insights into how cell death influences fibrosis progression and liver failure [18].

This paper describes a method that enables PI staining to be applied to liver tissue. Our technique allows the combination of PI with classical immunofluorescence, thus enabling the setup of multiplex staining in liver tissue.

## 2. Results

### 2.1. Co-Localization of PI and DAPI in Frozen Mouse Liver Slices

**Figure 1:**
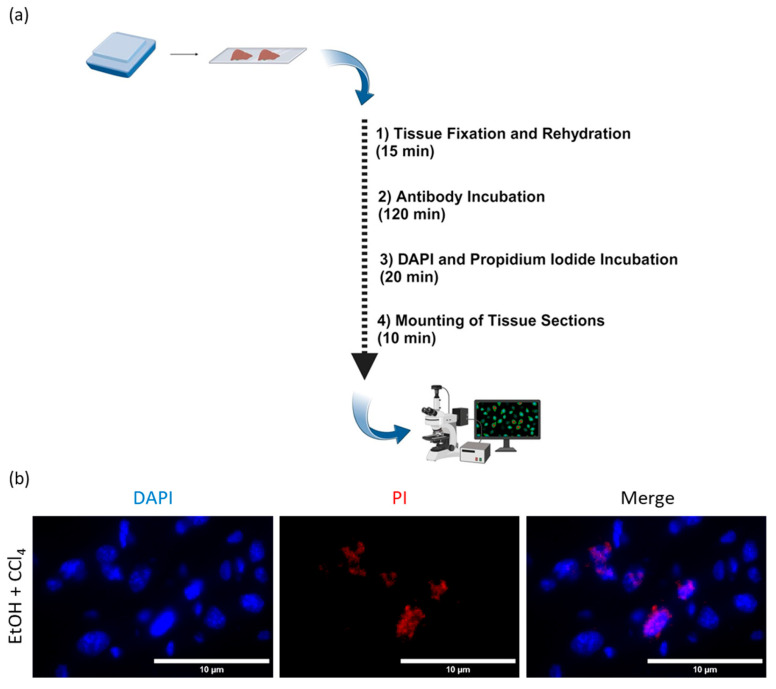
Propidium iodide (PI) and DAPI showed nuclear co-localization. (**a**) Workflow of PI staining protocol. (**b**) Representative image taken at 60× magnification from liver mouse cryosections and stained with PI (red) and DAPI (blue) showing nuclear co-localization. Scale bar = 10 μm.

To visualize the dead or dying cells within the frozen liver tissue we have developed fluorescence staining of PI (Figure 1a). This protocol can be performed in less than 1 h (without antibody incubation) or within less than 3 h if one chooses to combine PI with antibody incubation. In order to accurately quantify dead or dying cells, PI must intercalate into the DNA within the nuclei, which should show double positivity for PI and DAPI, as indicated by co-localization within the nuclei. To verify the exact overlap of PI and DAPI in the cell nuclei, frozen liver tissue sections were stained and analyzed under 60× magnification (Figure 1b). The high magnification allows detailed detection and evaluation of the staining patterns. Our results showed that PI and DAPI could be stained together, allowing accurate identification of dead or dying cells in frozen liver sections.

To investigate the potential application of PI staining in freshly frozen liver tissue sections and for benchmarking, we compared our protocol with the well-established and widely used TUNEL assay to identify dead or dying cells.

### 2.2. Comparison of PI and TUNEL Staining in Mouse Liver Sections

**Figure 2:**
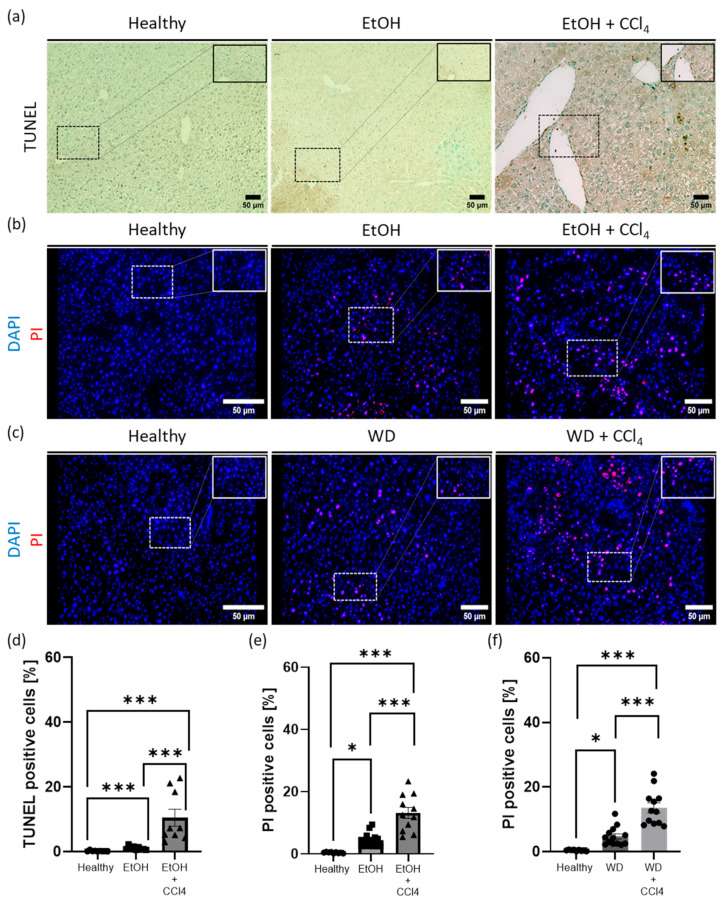
Fluorescence PI staining in liver mouse cryosections is comparable to the TUNEL assay. (**a**,**d**) Representative images from immunohistochemical TUNEL assay staining in paraffin-embedded liver sections from healthy, ethanol- (EtOH), and EtOH + CCl_4_-treated mice (n = 9) and their respective quantification analysis of TUNEL-positive cells (%). (**b**,**e**) Fluorescence staining of PI (red) and DAPI (blue) in liver cryosections of healthy, EtOH-, and EtOH + CCl_4_-treated mice (n = 9) and their respective morphometric analysis (%). (**c**,**f**) Representative fluorescence images of PI (red) and DAPI (blue) staining in liver cryosections of healthy, western diet- (WD), and WD + CCl_4_-treated mice (n = 9) and their quantification of PI positive cells (%). All images were taken at 20× magnification, scale bar = 50 μm. Results are expressed as mean ± SEM; * *p* < 0.05; *** *p* < 0.001.

Immunological detection of dying cells using the TUNEL assay was performed on paraffin-embedded sections from a mouse model of alcoholic liver disease (Figure 2a). Representative microscopic images were taken, and morphometric analysis was performed, which showed that the number of TUNEL positive cells was minimal (less than 1.5%) in both the healthy and ethanol-treated groups. However, in the fibrotic group (ethanol + CCl_4_), the number of moribund cells increased significantly to about 11% (Figure 2d).

In parallel, PI fluorescence staining was performed on the same frozen liver tissue sections (cryosections). Microscopic images of different liver cryosections showed a significant increase in dying cells over the course of the disease, comparable to the number quantified by the TUNEL assay (Figure 2b,e). We further validated our PI fluorescence staining protocol in another mouse model of liver disease (Figure 2c,f). In this model, mice were fed a high-fat, high-cholesterol diet (western diet, WD) to induce fatty liver disease, with some also receiving CCl_4_ injections (intraperitoneally) to induce liver fibrosis (Section 4). Consistent results were observed in both the alcoholic liver tissue cryosections and the western diet model cryosections.

Overall, our results show that fluorescence PI staining in freshly frozen mouse liver tissue provides quantitatively comparable results to the TUNEL assay in paraffin-embedded liver sections. These results show that fluorescence PI staining is a reliable method for quantifying dying cells in frozen liver tissue.

### 2.3. Co-Staining with Fluorescently Labelled Markers

For precise phenotyping of the respective cell type in a cell cluster, it is important to determine the exact cellular boundaries and thus be able to draw conclusions about the size and shape of the respective cell. To further explore the potential of co-staining with PI, we used the well-known fluorescent marker phalloidin, a bicyclic peptide from Amanita phalloides that specifically binds to actin filaments and accumulates in cell membranes.

**Figure 3:**
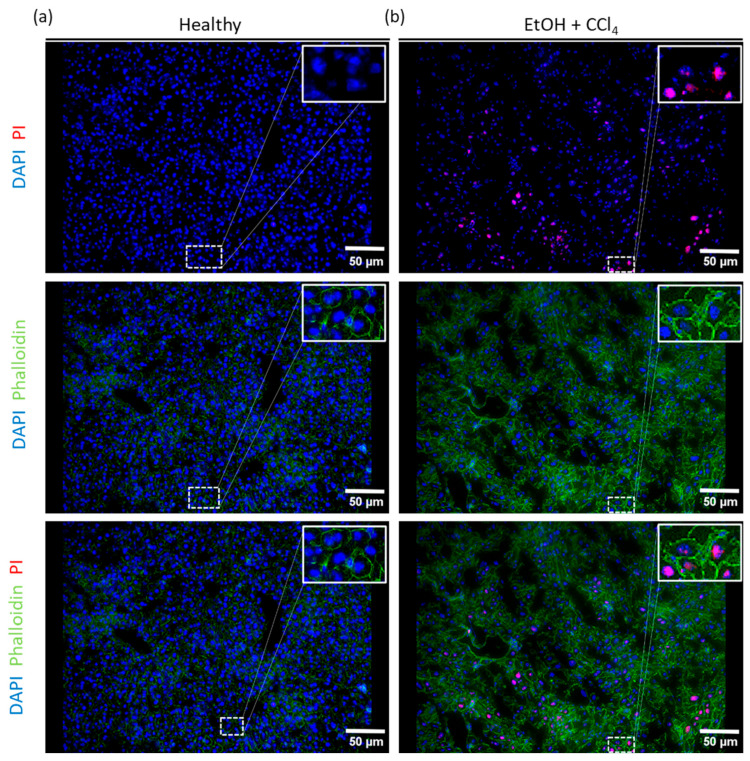
Fluorescence-labeled phalloidin co-stained with PI. (**a**) Representative fluorescence images from healthy liver mouse cryosections stained with PI (red), DAPI (blue), and phalloidin labeled with Alexa 488 (green). (**b**) Representative images from liver sections from mice treated with EtOH + CCl_4_ stained with PI (red), DAPI (blue), and phalloidin-Alexa488 (green). Images were taken at 20× magnification, scale bar = 50 μm.

Cryosections of healthy mouse liver tissue were stained according to the standard protocol (Section 4) with DAPI and PI alone and in a separate set with DAPI, PI, and fluorescently labelled phalloidin (Figure 3a, left panel). As expected, PI did not stain the healthy tissue, while phalloidin successfully labelled the actin filaments in the cell membranes. In contrast, liver cryosections from mice treated with ethanol and CCl_4_ to induce liver fibrosis showed a marked increase in PI-positive cells, indicating the presence of dying cells (Figure 3b, right panel). Phalloidin staining of actin filaments was consistent with the results from healthy tissue. These results show that PI staining can be used in conjunction with fluorescent markers in frozen liver tissue sections, providing information not only on the proportion of dying cells but also on their spatial organization in the tissue.

Additional insight into the spatial organization of dead or dying cells could be gained by using fluorescent markers that target specific cell structures.

### 2.4. Co-Staining with Fluorescently Labelled Antibodies to Identify Disease-Specific Zonation Patterns

During liver fibrosis, excessive activation of hepatic stellate cells (HSCs) leads to collagen accumulation and their transdifferentiation into myofibroblasts, which play a key role in liver fibrosis. Many studies have investigated liver fibrosis to better understand the progression of the disease [19,20], which is why we tested the applicability of PI co-staining with fluorescently labelled collagen [19].

**Figure 4:**
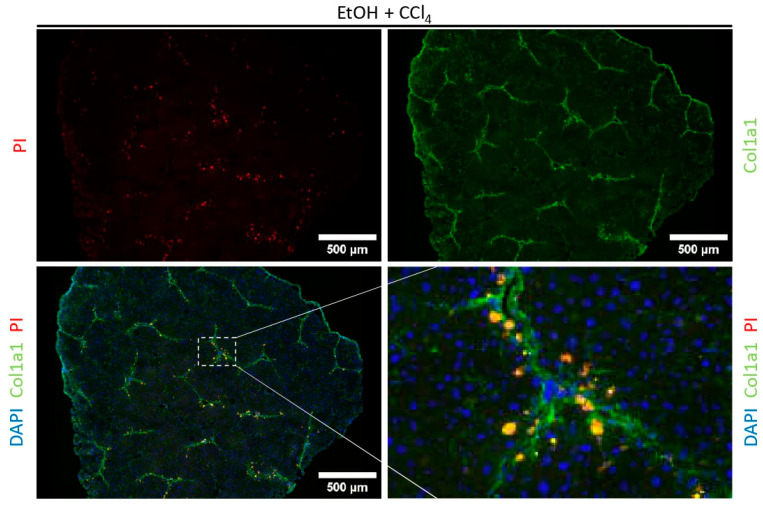
Co-staining of PI with a fluorescence-labeled collagen antibody. Collagen antibody is conjugated to Alexa Fluor 488 (green) and was co-stained with PI (red) and DAPI (blue) in liver cryosections of mice treated with EtOH + CCl4. Collagen1a1 (Col1a1) stained the fibrotic tissue characteristic from these treated mice. Merge image and the augmentation showed the region in the liver tissue where the cells are positive for PI and Col1a1 (yellow). Images were taken at 20× magnification, scale bar = 500 μm.

While fluorescence PI staining identifies dead or dying cells, the zonal localization of dying cells is crucial for understanding the pathobiology of different liver diseases. To determine the zone in which most cells die, we combined PI and DAPI staining with a fluorescently labelled antibody against collagen1a1 (Figure 4). In cryosections of mice treated with ethanol and CCl_4_ to induce fibrotic liver injury, simultaneous staining with PI and collagen1a1 showed the dead or dying cells mainly located near the collagen fibers. These results suggest that many of the dying liver cells are localized in spatial proximity to HSCs, the main producers of collagen, and that our protocol can be used to characterize both cell death and cellular localization patterns in parallel.

## 3. Discussion

In recent years, more and more methods have been developed to visualize multiplexed mRNA or proteins at the tissue and subcellular levels and to analyze them in the context of their spatial expression. The disadvantage of this development is the increasing need for expensive devices or the use of expensive kits, which are commercially advertised by the various providers. This is accompanied by a loss of the diversity of possible experiments, as highly specific adaptations and extensions of the commercial panels are carried out on a customer-specific basis, thus driving up the price even further. However, it is often sufficient to stain pathways with single specific proteins and characterize them in the context of their localization or cell type to study the progression of a disease or the effectiveness of a therapy. For most organs, doctors differentiate between acute and chronic organ failure. In the liver, which is primarily responsible for the metabolic detoxification of our entire body, cell death occurs in the clinical course of failure [21]. In the context of preclinical studies to understand the course of the disease or to test new therapeutic concepts, a cost-efficient and sensitive method for detecting dying cells is therefore required.

In this study, we demonstrated the utility of incorporating PI into a multiplex immunofluorescence staining protocol for membrane bound proteins in liver tissue. PI, a commonly used nucleic acid stain that selectively penetrates cells with damaged membranes, serves as an important marker of cell death and allows accurate identification of necrotic (dead) or late apoptotic cells (dying cells) [22]. Staining with PI is quick and easy to perform and offers more qualitative information and financial advantages compared to classical immunohistochemical-TUNEL staining [23]. In addition, the detection of cell death with PI in multiplex staining offers further advantages, especially in the study of cellular communication, tissue zonation and disease pathology in complex tissue environments [24]. It is important to note that this protocol does not allow permeabilization, as the specificity of PI is lost and can no longer be used to detect dead cells. However, this method also offers great advantages, particularly in terms of speed, simplicity of information acquisition, and cost efficiency. Especially in laboratories with limited financial resources, this method offers the opportunity to investigate cell death mechanisms in liver tissue without the use of expensive antibodies. In combination with the detection of extracellular proteins or membrane-bound markers, this method allows the investigation of intoxications or tissue changes in spatial resolution. Novel evaluation methods based on the spatial quantification of fluorescent proteins provide insights that were previously difficult to obtain with conventional histology [24,25].

A major advantage of PI staining in this context is its ability to identify dead or dying cells in specific tissue regions, which enables a deeper understanding of the disease pathobiology and the related cell–cell interactions in the vicinity of cell death. In liver tissue, for example, the localization of PI-positive cells can highlight areas of tissue injury or stress that may correlate with disease-specific zoning patterns. By using liver disease-specific markers, such as the extracellular matrix (e.g., collagen) or membranous protein expression (e.g., actin filaments), to delineate tissue zonation, the integration of PI enables a more comprehensive analysis of how cell death contributes to changes in zonal architecture and function. This is crucial to understand how regional damage in the liver affects overall tissue homeostasis and disease progression, e.g., in liver fibrosis progression or development of hepatocellular carcinoma [26].

In addition, PI staining in a multiplex immunofluorescence approach could enable parallel detection of immune cells in the surrounding tissue microenvironment, providing valuable insights into the local immune responses [23]. By simultaneously staining with immune cell markers, such as CD45, CD68, and specific cytokines, researchers can observe the recruitment and activation of immune cells in the environment of dying cells, providing additional insight into the role of (systemic) inflammation in modulating tissue homeostasis [27]. This is particularly important in chronic inflammatory diseases, where immune-mediated damage and repair processes occur in parallel with cell turnover and tissue remodeling [28,29].

Furthermore, the ability to identify immune cell infiltration along with markers of tissue damage and cell death provides important information about the impact of (systemic) inflammation and the efficacy of therapeutic interventions [30]. By observing how immune responses and mechanisms of tissue damage are spatially related, researchers can also accurately assess how well immunomodulatory treatment strategies block specific pathomechanisms. This dual focus is crucial for the development of optimized therapeutic approaches that increasingly aim to alleviate systemic inflammatory mechanisms while preserving or restoring tissue integrity [31,32].

In summary, the inclusion of PI in multiplex immunofluorescence staining greatly enhances our ability to analyze complex tissue environments by enabling the simultaneous detection of cell death, immune responses and tissue-specific zonation. This approach provides crucial insights into disease mechanisms and offers a powerful tool to evaluate the efficacy of novel therapeutic strategies aimed at restoring tissue homeostasis and attenuating inflammatory damage.

## 4. Materials and Methods

### 4.1. Preparation of PI Solution

To create a 1.5 mM stock solution, 1 mg of PI (Fluka, Buchs, Switzerland) (Table 1) was dissolved in 1 mL of deionized water. To achieve the desired working concentration (0.5 µM), the stock solution 1:3000 was diluted in 1× phosphate-buffered saline (PBS) (AppliChem GmbH, Darmstadt, Germany). The dissolved PI (Fluka) was stored at 4 °C, keeping it protected from light.

### 4.2. Animal Models of Chronic Liver Disease

A total of 45 mice were used for this study. Male wild-type (WT, C57BL6/J) mice (10 weeks old) were purchased (Charles River Laboratories Research Model and Services Germany, Sulzfeld, Germany). The experiments were performed according to the guidelines and regulations approved by the Regierungspräsidium Darmstadt, the responsible committee for animal studies in the German federal state of Hesse (permission number FK/2003). Steatosis was induced by the addition of ethanol to the drinking water (4% during week 1, 8% during week 2, and 16% until animals were sacrificed) and normal chow (Ssniff, Soest, Germany) to induce alcoholic steatohepatitis or by an additional high-fat, cholesterol-rich diet without ethanol (WD; Ssniff) to induce metabolic dysfunction-associated steatohepatitis. Liver fibrosis was induced by a 2 µL/g CCl_4_ injection (intraperitoneal; CCl_4_:Corn oil = 1:2) 2 times a week for 7 weeks. These models are described in detail in the following papers [19,33,34,35,36]. Mice were anaesthetized with a mixture of ketamine (Ketaset, 100 mg/mL; Zoetis, Berlin, Germany) and xylazine (Xylazin, 20 mg/mL; WDT, Garbsen, Germany). Cervical dislocation took place directly after the mice were under anesthesia.

#### 4.2.1. Tissue Fixation and Rehydration

After harvesting, the organs were immersed in Optimal Cutting Temperature embedding medium (Science Services, Munich, Germany), placed in dry ice, and stored at −80 °C. The tissue was placed at −14 °C in the microtome (Leica CM 1900 UV Research Cryostat) and sectioned into 5-µm thin slices. The slides were mounted on superfrost slides (Epredia), dried at room temperature for 30 min, and stored at −80 °C until further use. For fixation, the frozen cryosections were immersed in −20 °C methanol (Sigma-Aldrich, Saint Louis, MO, USA) for 2 min, followed by −20 °C acetone (Sigma-Aldrich, Saint Louis, MO, USA) for an additional 20 s in the staining boxes (Carl Roth, Karlsruhe, Germany). After fixation, the Optimal Cutting Temperature embedding medium (Science Services) was carefully removed using tweezers. A hydrophobic barrier was then drawn around the tissue using a DAKO-pen (DAKO North America Inc., Nowy Sącz, Poland) to contain reagents during subsequent staining steps. The barrier was allowed to dry completely before proceeding. The tissue sections were then rehydrated by placing them in 1× PBS (AppliChem GmbH) at room temperature for 10 min in staining boxes (Carl Roth).

#### 4.2.2. Antibody Incubation

After rehydration, the sections were blocked with 1% bovine serum albumin (AppliChem GmbH, Darmstadt, Germany) in 1× PBS (AppliChem GmbH) for 45 min to reduce non-specific antibody binding. Following the blocking step, the blocking solution was removed, and the tissue sections were incubated with phalloidin A488 (Invitrogen, Carlsbad, CA, USA), 1:1000 diluted in 1% bovine serum albumin (AppliChem GmbH) or with pre-labeled anti-col1a1 (Alexa488) (Cell Signaling Technology, Danvers, MA, USA), or 1:1000 diluted in 1% bovine serum albumin (AppliChem GmbH). The sections were incubated with the antibody for 1 h at room temperature to ensure adequate binding. After phalloidin (Invitrogen) or antibody incubation, the tissue sections were thoroughly washed to ensure the removal of any unbound antibodies. The washing process involved two 5-min washes with 1× PBS (AppliChem GmbH) containing 0.05% Tween 20 (Sigma-Aldrich, Saint Louis, MO, USA), followed by an additional 5-min wash with 1× PBS (AppliChem GmbH) alone. This step was critical to remove any residual blocking solution and non-specifically bound antibodies, ensuring clear staining results.

#### 4.2.3. DAPI and PI Incubation

The tissue was incubated with 1 µM DAPI (Abcam, Cambridge, UK) for 5 min to stain the nuclei. DAPI (Abcam) binds strongly to DNA, providing a distinct blue fluorescence that allows for the identification of all cell nuclei in the tissue section. After DAPI (Abcam) incubation, the tissue was washed three times with 1× PBS (AppliChem GmbH), with each wash lasting 5 min, to remove excess DAPI (Abcam) and reduce background fluorescence.

Subsequently, the tissue sections were stained with 0.5 µM PI (Fluka) diluted in 1× PBS 1× (AppliChem GmbH). PI (Fluka) was pipetted onto the tissue and incubated in a dark chamber for 10 min at room temperature to preserve the fluorescence. Finally, the tissue was washed by immersing it in 1× PBS (AppliChem GmbH) three times in staining boxes, with each immersion lasting 30 s. Washing longer than 30 s each time leads to a decrease in PI signal intensity.

#### 4.2.4. Mounting of Tissue Sections

Finally, the tissue sections were mounted using DAKO Fluorescent Mounting Medium (DAKO North America Inc., Nowy Sącz, Poland) to preserve fluorescence and prevent photobleaching during microscopy analysis. The DAKO Fluorescent Mounting Medium (DAKO North America Inc.) was dried completely before the tissue was observed under the microscope.

#### 4.2.5. Imaging and Quantification

The stained tissue sections were visualized using a BZ-X800 fluorescence microscope (Keyence, Ōsaka, Japan) with a 20× or 60× magnification and analyzed with QuPath Version 0.5.1 [37]. The software was designed for annotation and visualization, featuring built-in algorithms for common tasks such as cell and tissue detection, along with interactive machine learning for object and pixel classification. Slide scans were loaded into QuPath, where all detected cells were annotated using DAPI (Abcam). The proportion of positive staining per scan was then calculated through pixel classification.

For quantification purposes, DAPI-positive cells were counted by running the command: “Analyze ‣ Cell detection ‣ Positive cell detection”. Further classification of cells based on fluorescence intensity was executed using the command: “Classify ‣ Object classification ‣ Set cell intensity classifications”. This helps to specifically identify PI-positive cells and to evaluate the extent of cell death.

#### 4.2.6. Statistical Analysis

Statistical analyses were conducted using GraphPad Prism version 10.3.1 Statistically significant differences across all experiments were determined using one-way analysis of variance (ANOVA), followed by Tukey’s post hoc correction. Each experiment was conducted at least three times. Statistical significance is represented as follows: * *p* < 0.05 and *** *p* < 0.001.

## 5. Frequently Asked Questions

- Can other chemicals be used instead of methanol and acetone to fixate the tissue?

Using other chemicals can affect the staining of PI. For example, we used 4% paraformaldehyde to fixate the tissue, but this had a negative effect on the staining. Fixation with paraformaldehyde can influence the liquid–liquid phase separation within the cells and thus possibly impair the staining of DNA with PI [38]. The fixation method should be selected carefully.

- Is it possible to use this protocol for paraffin sections?

We also tested the staining in paraffin sections, but it did not work there. This could also be due to the pre-treatment with paraformaldehyde.

- Can the protocol also be used in organs other than the liver?

**Figure 5:**
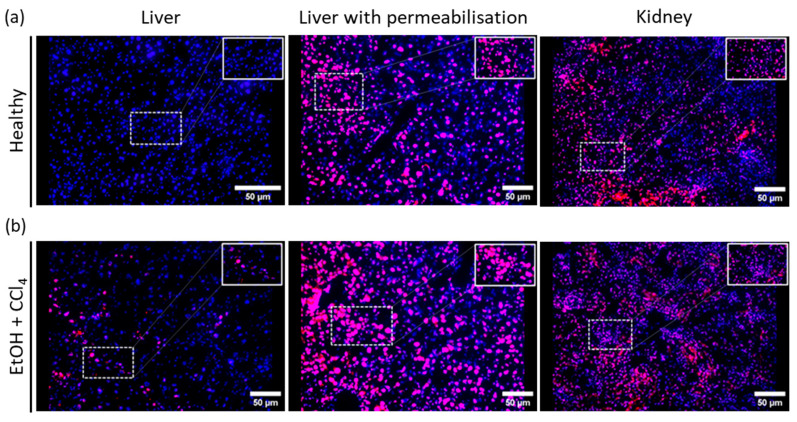
Tissue permeabilization increases PI-positive staining. (**a**,**b**) Liver cryosections from healthy and EtOH + CCl_4_ mice, respectively, were permeabilized with 0.1% Triton x-100 and stained afterwards with PI (red) and DAPI (blue). The number of PI-positive cells after permeabilization significantly increased when compared to the untreated ones. Kidney cryosections from mice (healthy and EtOH + CCl_4_) were stained with PI and showed high number of PI-positive cells even in healthy group. Images were taken with 20× magnification, scale bar = 50 μm.

The protocol was also carried out in cryosections of the kidney, but this resulted in non-specific staining of many cells, so that it was no longer possible to distinguish between intact or dead/dying cells (Figure 5a,b). This problem can be explained by the high permeability of the kidney [39]. We cannot yet say whether the protocol also works in organs other than the liver. It is therefore advisable to use this protocol with caution outside the liver. Further optimization may be necessary for other tissues.

- Can I use PI staining multiplexed with cytosolic/nuclear proteins?

Unfortunately, it is not possible to stain cytosolic and/or nuclear proteins with this protocol, as the tissue must be permeabilized using detergents (e.g., Tween 20, Triton-X100). Antibodies that require permeabilization cannot enter the cells and will produce an unspecific staining pattern. Additionally, after permeabilization, PI will stain any nuclei positive (Figure 5a,b).

## Figures and Tables

**Table 1:** Reagents and resources that were used for this study together with their source and identifier.

Reagent or Resource	Source	Location	Identifier
4’,6-Diamidin-2-phenylindol (DAPI)	Abcam	Campbridge, United Kingdom	Ab228549
Acetone p.a.	Sigma-Aldrich	Saint Louis, MO, USA	32201-1L
Alexa Fluor 488 phalloidin	Invitrogen	Carlsbad, CA, USA	A12379
Anti-col1a1 pre-labeled (Alexa488)	Cell Signaling Technology	Danvers, MA, USA	28368S
Bovine serum albumin (BSA)	AppliChem GmbH	Darmstadt, Germany	A1391, 0100
BZ-X800 fluorescence microscope	Keyence	Ōsaka, Japan	
DAKO Fluorescent Mounting Medium	DAKO North America Inc.	Nowy Sącz, Poland	S3023
DAKO-pen	DAKO North America Inc	Nowy Sącz, Poland	N71310-N
Distilled water			
Ketamine (100 mg/mL)	Zoetis	Berlin, Germany	40019397
Methanol p.a.	Sigma-Aldrich	Saint Louis, MO, USA	32213-2.5-M
Optimal Cutting Temperature embedding medium	Science Services	Munich, Germany	SA62550-01
Phosphate-buffered saline (PBS)	AppliChem GmbH	Darmstadt, Germany	A0964, 9050
Propidium iodide (PI)	Fluka	Buchs, Switzerland	81845
Staining box	Carl Roth	Karlsruhe, Germany	HA44.1
Superfrost slides	epredia	Basel, Switzerland	J1800AMNZ
Tween-20	Sigma-Aldrich	Saint Louis, MO, USA	P1379-500ML
Xylazin (20 mg/mL)	WDT	Garbsen, Germany	04-03-9296/01

## Data Availability

All data are available in the main text.

## Abbreviations

Terms and their abbreviations used in this paper.

4′,6-diamidino-2-phenylindole	DAPI
Collagen1a1	Col1a1
Ethanol	EtOH
Hepatic stellate cell	HSC
Phosphat buffered saline	PBS
Propidium iodide	PI
TdT-mediated dUTP-biotin nick end labeling	TUNEL
Tetrachlormethane	CCl_4_
Western diet	WD
